# Findings regarding emotion regulation strategies and quality of life's domains in families having children with spinal muscular atrophy

**DOI:** 10.25122/jml-2021-0098

**Published:** 2021

**Authors:** Maria Veronica Morcov, Liliana Padure, Cristian Gabriel Morcov, Gelu Onose

**Affiliations:** 1. Dr. N. Robanescu National Clinical Center of Neurorehabilitation for Children, Bucharest, Romania; 2. Faculty of Medicine, Carol Davila University of Medicine and Pharmacy, Bucharest, Romania; 3. The Neuromuscular Rehabilitation Clinic Division, Bagdasar-Arseni Clinical Emergency Hospital, Bucharest, Romania

**Keywords:** family, spinal muscular atrophy, emotion regulation, quality of life

## Abstract

The severity of motor impairment and the psycho-emotional and social consequences of spinal muscular atrophy (SMA) impact both children and their families, who must adapt using cognitive-emotional strategies. We aimed to determine whether the domains of quality of life and the consequent emotion regulation strategies could be related, and if so, to what quantitative, at a statistically significant level. This study was conducted at the Dr. N. Robanescu National Clinical Center of Neurorehabilitation for Children and included 33 mothers questioned using the PedsQL-Family Impact Module (PedsQL-FIM) and Cognitive Emotion Regulation Questionnaire (CERQ). Statistical analysis of PedsQL-FIM data showed high positive Spearman’s rho correlations between communication and social functioning (p=0.719), daily activities and cognitive functioning (p=0.704), family relationships and daily activities (p=0.705). The analysis of the Spearman’s rho correlation coefficients reflected some moderate positive correlations between CERQ subscales: self-blame and catastrophizing (p=0.577), acceptance and refocus on planning (p=0.577), acceptance and putting into perspective (p=0.532), refocus on planning and positive reappraisal (p=0.630), positive reappraisal and putting into perspective (p=0.609). Maladaptive strategies affect family relationships, cognitive and social functioning, emotional functioning, and communication inside the family. Positive correlations were found between the adaptive strategies (acceptance, refocus on planning, putting into perspective, positive reappraisal) in the participants within our study group, showing their interest in attitude changing and actively solving the family tasks related to children’s illness.

## INTRODUCTION

Spinal muscular atrophy (SMA) is a rare autosomal-recessive neuromuscular disease [1, 2] characterized by progressive muscle atrophy and weakness [3] that causes severe physical disabilities in the affected individuals and a significant burden on their kin, thus affecting the entire family’s quality of life [4].

Although research regarding SMA disease continues and new classifications appear, currently four subtypes of SMA have been described in the literature, based on the time of onset and severity:

- Type I, also called Werdnig-Hoffman disease, is the most common SMA subtype that appears within the first 6 months of life. It is characterized by severe generalized weakness, hypotonia, impaired respiratory function, impaired feeding and swallowing difficulties [5–9];
- Type II, known as Dubowitz disease, can be diagnosed in children aged between 6–18 months. These children can reach the sitting motor stage without support but cannot reach the stage of independent walking [6–9];
- Type III, also called Kugelberg-Welander disease or juvenile SMA, involves symptoms that can be diagnosed after 18 months. Even if they have balance difficulties, children become able to walk without help [6–9];
- Type IV, also called adult-onset SMA, is defined by walking with difficulty, weakness of upper and lower limbs [6, 8, 9].

Type 0, which is considered the most severe type, was added to the four types of SMA described above [9]. The onset is in the intrauterine period and reduced fetal movements could be observed. According to data from the literature, newborns diagnosed with type 0 live up to a maximum of 6 months [10]. The severity of motor impairment and the psycho-emotional and social implications of the first three types of SMA defined above impact on both children and/or adolescents and their families who must continuously adapt using different cognitive-emotional strategies. After searching the literature, we found that it provides little data about the relationship between the disease (including our interested pathology – SMA) and the possible ways the family responds in the sense of adapting or not to the burden caused by it.

Few studies were conducted on emotion regulation in adult patients with neurodegenerative diseases [11], but even fewer have been found for families with children and/or adolescents with SMA. Emotion is an internal or external signal that determines the individual’s adaptation to the environment through a process called emotional regulation [12]. When people are confronted with unfavorable living conditions, they can modify the intensity of their emotions through cognitive, voluntary, or beyond their control processes. Emotional regulation strategies can be “healthy” or “unhealthy” [13]; the healthy ones help to establish interpersonal and social relationships and achieve a better quality of life, leading to equilibrium in a family with a disabled child.

Besides the medical (pharmacological and physical-rehabilitative) treatment used to reduce symptoms’ severity of this disease, identifying the emotional regulation strategies used by families with children and/or adolescents with SMA can also help specialists to find and offer solutions for better family psychological and social assistance. Through an analysis of the families with children and/or adolescents with SMA, we aimed to find the answer to the question of whether the quality of life’s domains and the consequent emotion regulation strategies can be related at a statistically significant level and, if so, to what quantitative.

## MATERIAL AND METHODS

The study was conducted at the Dr. N. Robanescu National Center of Neurorehabilitation for Children and included 33 mothers of children and/or adolescents with SMA. They were questioned using the two largely accepted related assessment scales: PedsQL-Family Impact Module (PedsQL-FIM) [14] and Cognitive Emotion Regulation Questionnaire (CERQ) [15], the Romanian versions. The first author has obtained agreement to use both scales. PedsQL-FIM, designed by the authors as a complex multidimensional tool, “a parent proxy-report instrument” [14], was initially used in families having children with chronic conditions. This instrument is composed of 36 items comprising 8 domains: “Physical Functioning” (6 items), “Emotional Functioning” (5 items), “Social Functioning” (4 items), “Cognitive Functioning” (5 items), “Communication” (3 items), “Worry” (5 items), “Daily Activities” (3 items), “Family Relationships” (5 items) [14], each item being evaluated on a 5-point Likert scale from 0 (Never a problem) to 4 (Almost always a problem). The higher score obtained by the sum of the reversed and linearly transformed score to a 0–100 scale (0=100, 1=75, 2=50, 3=25, 4=0) of the items for each domain, indicates better functionality [14].

The Cognitive Emotion Regulation Questionnaire [15] is a 36-item questionnaire containing nine subscales which include the next used strategies by a person: “self-blame”, “acceptance”, “rumination”, “positive refocusing”, “refocus on planning”, “positive reappraisal”, “putting into perspective”, “catastrophizing”, and “blaming others”/“other-blame”. The 36 items are divided into groups of four, resulting in nine strategies that propose evaluating cognitive emotion regulation strategies (adaptive and maladaptive) in response to a specific situation that a person experiences at some point in life [16]. According to the literature, positive changes (adaptive strategies) are given by “acceptance”, “positive refocusing”, “refocus on planning”, “putting into perspective” and “positive reappraisal” [17–19]. The negative changes (maladaptive strategies) are given by “self-blame”, “rumination”, “catastrophizing” and “other-blame” [17–19].

On the other hand, acceptance was “more strongly related to less adaptive strategies latent factor” [19], considering that “the presumably adaptive role of acceptance needs to be reconceptualized” [20]. Cognitive emotion regulation strategies were also measured on a 5-point Likert scale ranging from 1 (almost never) to 5 (almost always). The sum of the items of each strategy is compared with the reference values, where high scores represent the dominant strategy/strategies chosen by the patient [21].

For statistical data processing, we have used Spearman’s rho correlations and Cronbach’s alpha coefficient, and the Statistical Package for the Social Sciences (SPSS) version 27. The value of the Spearman correlation coefficient can range from –1 to 1, indicating the strength of the relationship between two variables [22–24]. Cronbach’s alpha coefficient determines if the scale is reliable and can range from unacceptable (0.5>α) to excellent (α>0.9) [25].

## RESULTS

Data on SMA onset (type) and family characteristics (residence areas, education level, marital status) were collected from 33 mothers enrolled in our study, as described above. Mothers of patients with SMA type 2 (39.3% of respondents) were the most common, followed by SMA type 1 and SMA type 3, which were evenly distributed (30.3%). 51.6% came from urban areas and 48.4% from rural areas. The mothers' education level was “high school or less” for 69.7% of the respondents and “college” (university) for 30.3% of them. In terms of marital status, 78.8% of respondents were married, while 21.2% were divorced – previously married or single persons.

Regarding PedsQL-FIM, the inner relationship between items, as resulted from our analysis, was assessed using Cronbach's alpha coefficient, the value of 0.896 showing a good internal consistency according to Cronbach's alpha classification [25]. For PedsQL-FIM, we also calculated reversed scores to find higher values corresponding to better functionality in each domain. Table 1 summarizes the results, which were calculated as a percentage among the 33 respondents. Regarding CERQ , the inner relationship between items, as resulted from our analysis, was also assessed using Cronbach's alpha coefficient, the value of 0.852 showing a good internal consistency according to Cronbach's alpha classification [25].

**Table 1:** Highest score frequency for each PedsQL-FIM domain.

PedsQL-Family Impact Module domains	Frequency* (% among 33 respondents)
**Physical Functioning**	3%
**Emotional Functioning**	3%
**Social Functioning**	6.1%
**Cognitive Functioning**	9.1%
**Communication**	9.1%
**Worry**	3%
**Daily Activities**	9.1%
**Family Relationships**	21.2%

* reflects the percentages of the best functionality (not a simplistic summation of percentages).

We also calculated CERQ scores to see what percentage of respondents chose a very high score in response to a specific strategy. Table 2 shows the results as a percentage calculated among the 33 respondents. The Spearman's rho correlation coefficients study reveals low and moderate positive correlations [23] between the PedsQL-FIM questionnaire domains, with a few high positive correlations, as shown in Table 3. Table 4 shows that Spearman's rho correlation coefficients reveal generally low [23] and some moderate positive correlations [23] between CERQ subscales. Low and moderate negative Spearman's rho correlations were found between PedsQL-FIM domains and CERQ subscales (Table 5) [23].

**Table 2:** Highest score frequency for each CERQ strategy.

CERQ Strategy	Frequency* (% among 33 respondents)
**Self-blame**	6.1%
**Acceptance**	24.2%
**Rumination**	12.1%
**Positive refocusing**	30.3%
**Refocus on planning**	15.2%
**Positive reappraisal**	15.2%
**Putting into perspective**	27.3%
**Catastrophizing**	18.2%
**Other-blame**	6.1%

* reflects the percentages of the best functionality (not a simplistic summation of percentages).

**Table 3:** Spearman’s rho correlations between PedsQL-FIM domains.

Spearman's rho	Physical Functioning	Emotional Functioning	Social Functioning	Cognitive Functioning	Communication	Worry	Daily Activities	Family Relationships
**Physical Functioning**	1.000	.679**	.501**	.361*	.512**	.402*	.435*	.505**
**Emotional Functioning**	.679**	1.000	.568**	.478**	.569**	.440*	.383*	.479**
**Social Functioning**	.501**	.568**	1.000	.677**	.719**	.484**	.559**	.671**
**Cognitive Functioning**	.361*	.478**	.677**	1.000	.485**	.286	.704**	.606**
**Communication**	.512**	.569**	.719**	.485**	1.000	.608**	.491**	.686**
**Worry**	.402*	.440*	.484**	.286	.608**	1.000	.290	.530**
**Daily Activities**	.435*	.383*	.559**	.704**	.491**	.290	1.000	.705**
**Family Relationships**	.505**	.479**	.671**	.606**	.686**	.530**	.705**	1.000

** Correlation is significant at 0.01 (2-tailed); * Correlation is significant at 0.05 (2-tailed).

**Table 4:** Spearman’s rho correlations between CERQ subscales.

Spearman's rho	Self-blame	Acceptance	Rumination	Positive refocusing	Refocus on planning	Positive reappraisal	Putting into perspective	Catastrophizing	Other-blame
**Self-blame**	1.000	.063	.368*	-.146	-.024	-.044	-.097	.577**	.407*
**Acceptance**	.063	1.000	.379*	.438*	.577**	.373*	.532**	.185	.176
**Rumination**	.368*	.379*	1.000	.194	.482**	.205	.240	.440*	.351*
**Positive refocusing**	-.146	.438*	.194	1.000	.424*	.365*	.415*	.100	.193
**Refocus on planning**	-.024	.577**	.482**	.424*	1.000	.630**	.478**	.229	.295
**Positive reappraisal**	-.044	.373*	.205	.365*	.630**	1.000	.609**	.053	-.037
**Putting into perspective**	-.097	.532**	.240	.415*	.478**	.609**	1.000	.071	.054
**Catastrophizing**	.577**	.185	.440*	.100	.229	.053	.071	1.000	.387*
**Other-blame**	.407*	.176	.351*	.193	.295	-.037	.054	.387*	1.000

** Correlation is significant at 0.01 (2-tailed); * Correlation is significant at 0.05 (2-tailed).

**Table 5:** Spearman’s rho correlations between PedsQL-FIM domains and CERQ subscales.

Spearman's rho(significance below)	Self-blame	Acceptance	Rumination	Positive refocusing	Refocus on planning	Positive reappraisal	Putting into perspective	Catastrophizing	Other-blame
**Physical Functioning**	-.272	.060	-.355*	.177	.205	.202	.062	-.073	-.052
**Emotional Functioning**	-.369*	-.143	-.549**	.103	-.064	.021	-.014	-.293	-.032
**Social Functioning**	-.448**	-.301	-.386*	.108	-.010	.172	.103	-.197	-.160
**Cognitive Functioning**	-.305	-.336	-.478**	.121	-.040	.061	-.100	-.111	-.061
**Communication**	-.502**	-.144	-.393*	.046	-.068	.111	.044	-.298	-.276
**Worry**	-.411*	-.105	-.292	.157	-.029	.209	-.043	-.160	-.304
**Daily Activities**	-.092	-.168	-.357*	.054	-.145	-.065	-.152	.054	.039
**Family Relationships**	-.492**	-.253	-.496**	.015	-.211	-.001	-.099	-.246	-.231

** Correlation is significant at 0.01 (2-tailed); * Correlation is significant at 0.05 (2-tailed).

## DISCUSSION

Statistical analysis of PedsQL-FIM data (Table 1) revealed that the “family relationships” domain had the highest frequency of the maximum value, accounting for 21.2 % of all respondents, who considered that they do not have “problems with family relationships, including communication, stress, and conflicts between family members, and difficulty making decisions and solving problems as a family” [14].

At the same time, only 3% of respondents scored with maximum value the “physical functioning”, “emotional functioning”, and “worry” domains (Table 1). According to their responses, they did not have “problems with physical functioning, including feeling tired, getting headaches, feeling weak, and stomach problems” [14], “problems with emotional functioning, including anxiety, sadness, anger, frustration, and feeling helpless or hopeless” [14] or “problems with worrying, including worrying about child’s treatments and side effects, about others’ reactions to child’s condition, about the effect of the illness on the rest of the family, and about child’s future” [14].

Statistical analysis of CERQ data (Table 2) showed that the highest score obtained by 30.3% of questioned mothers was for “positive refocusing”. Our respondents use “positive refocusing” as an adaptive strategy, thinking of “nicer things” and “pleasant experiences”, considering that it is essential to “think of something nice instead of what has happened” [21]. “Putting into perspective” (27.3% from respondents) and “acceptance” (24.2% from respondents) were the next strategies with a “very high” score among our respondents (Table 2). Acceptance of disease and search for solutions to improve family’s condition were viewed as valuable strategies for better outcomes in children and/or adolescents with SMA and their families. According to the findings (Table 2), the maximum values for “self-blame” and “other-blame” were 6.1% for both strategies. 93.9% of the respondents believed that others could not be blamed for what happened to them, 60.6% of them assigned a low score to the “other-blame” strategy.

The analysis of Spearman’s rho correlation coefficients (Table 3) generally reflects the moderate positive correlations [23] between the domains of the PedsQL-FIM questionnaire. Because caring for a child with SMA changes the “whole life” of the family [26], we can say that the greater the emotional stability of the members, the better the physical and social functioning and communication. There are also few high positive correlations [23] (Table 3) between the domains of the PedsQL-FIM questionnaire. For our respondents, communication, including the understanding of the family’s situation by others, discussing the child’s health condition, and communicating with health professionals, is highly correlated with social functioning, which includes feeling isolated or not, receiving support from others, and finding time or energy for social activities.

The capacity to adequately perform domestic duties is highly linked to family harmony and communication (Table 3). Household activities do not require additional effort when cognitive functions, expressed by focusing attention, the ability to store and update information, and the ability to think quickly, are good (Table 3).

The analysis of Spearman’s rho correlation coefficients (Table 4) generally reflects low positive correlations [23] between CERQ sub-scales. Low positive correlations between subscales suggest that they are relatively independent. However, there are some moderate positive correlations (Table 4) between CERQ subscales [23].

We found moderate positive correlations between two maladaptive strategies (catastrophizing and self-blame) that we identified in the literature as anxiety-related strategies [17]. Thus, we appreciate that the correlation between the two strategies could be caused by the stress due to the severity of the disease in families with SMA children. The moderate positive correlations (Table 4) found between the adaptive strategies (acceptance, refocus on planning, putting into perspective, positive reappraisal) in our study group might show the interest of the enrolled participants in behavior shifting and actively solving the family challenge represented by their children’s/ adolescent’ sickness and consequent disability. Disease acceptance is considered one of the “predictors of subjective well-being among individuals with a chronic physical or psychological disability” [27] and a first step for the family to find ways of improving its quality of life by planning and seeking therapeutic solutions for children/adolescents.

The analysis of Spearman’s rho correlations between PedsQL-FIM domains and CERQ subscales revealed negative correlations of low and moderate strength. The results of our study suggest that decreased emotional functioning can be associated with more intense rumination (see Table 5). Among our study participants, the communication domain has a moderate negative correlation with the self-blame strategy (Table 5), suggesting that mothers could feel guilty about their child’s illness when others do not understand the family’s situation, have difficulty talking about the child’s health condition, and communicate with health professionals. Our findings (Table 5) suggest that adequate family relationships can reduce their members’ self-blame and rumination to a small extent. Mothers’ responses (Table 5) show that their thoughts and concerns about their child’s condition have little impact on cognitive functioning issues, including problems paying attention, remembering things, and thinking rapidly. Our study found a low negative correlation between social function and self-blame (Table 5), which could indicate that responsibility for the child’s disease and social relationships had only a minor relation.

## CONCLUSIONS

SMA is a rare autosomal-recessive neuromuscular disease that causes significant physical disabilities in affected individuals as well as a significant burden on their families. The medical, psychological and social implications of SMA affect both children, adolescents and their families, who must constantly adapt using various cognitive-emotional strategies. Moderate correlations of maladaptive strategies used by mothers within our research could be caused by the stress due to the severity of the disease in families with SMA children and/ or adolescents.

The positive correlations found between the adaptive strategies (acceptance, refocus on planning, putting into perspective, positive reappraisal) in our study group might indicate moderate interest in looking for solutions to improve the quality of life of SMA children and/or adolescents and their entire families. The results of this research, especially if underpinned on a bigger statistical power in the future, could help specialists in providing psychological support for families of SMA children and/or adolescents.
